# Comparative analysis of sclerotherapy and surgical resection for pediatric pyogenic granuloma: a retrospective cohort study

**DOI:** 10.3389/fped.2026.1782257

**Published:** 2026-03-20

**Authors:** Zhenxiang Yuan, Wei Yao, Kai Li, Zuopeng Wang

**Affiliations:** Department of Pediatric Surgery, Children’s Hospital of Fudan University, National Children’s Medical Center, Shanghai, China

**Keywords:** patient observer scar assessment scale, pyogenic granuloma, sclerotherapy, surgical excision, treatment

## Abstract

**Background:**

Pyogenic granuloma (PG) is a benign vascular lesion frequently encountered in children and young adults. Although surgical excision is traditionally considered the standard of care, it may be limited by operative complexity, postoperative scarring, and patient discomfort. Sclerotherapy with polidocanol has recently emerged as a minimally invasive alternative, but direct comparative data in pediatric populations remain limited.

**Methods:**

We conducted a retrospective cohort study of 30 pediatric patients diagnosed with PG and treated at our institution between January 2022 and February 2023. Fifteen patients underwent surgical excision and fifteen underwent intralesional sclerotherapy with 1% polidocanol. Clinical outcomes, recurrence rates, complications, and scar quality were evaluated. Scar assessment was performed using the Patient and Observer Scar Assessment Scale (POSAS 2.0) by both parents and two independent blinded observers. Statistical analysis included independent t-tests, *χ*^2^ tests.

**Results:**

Complete resolution was achieved in all patients (overall cure rate: 100%) with no recurrence during follow-up. In the sclerotherapy group, 93.3% achieved complete resolution after a single session, with one patient requiring a second session. The mean operative duration was significantly shorter for sclerotherapy compared with surgery (*p* < 0.05). Postoperative scarring was observed in 66.7% of the surgical group but only 6.7% of the sclerotherapy group (*p* < 0.01). POSAS scores for both patient and observer scales were significantly lower (indicating better cosmetic outcome) in the sclerotherapy cohort (*p* < 0.01). No major complications occurred.

**Conclusion:**

Both surgical excision and polidocanol sclerotherapy are highly effective for treating pediatric PG. However, sclerotherapy offers advantages including reduced scarring, shorter procedure time, and superior cosmetic results, suggesting it may be considered a first-line treatment. Larger prospective randomized trials are warranted to confirm these findings and optimize patient selection criteria.

## Introduction

Pyogenic granuloma (PG), also referred to as lobular capillary hemangioma, granuloma telangiectaticum, or granuloma gravidarum, is a common benign vascular tumor of the skin and mucous membranes (1). It predominantly affects children, young adults, and pregnant women. In its early stage, PG typically presents as a small, bright-red to dark-red papule without significant subjective symptoms. Over time, the lesion gradually—or sometimes rapidly—enlarges, forming a pedunculated or sessile nodule with an eroded surface and a marked tendency to bleed. Lesions are usually less than 2 cm in diameter and most commonly occur on the head, neck, face, trunk, and extremities.

The precise pathogenesis of PG remains incompletely understood (2). Recurrence is a frequent clinical challenge, often attributed to incomplete excision (residual disease) or persistent triggering stimuli (3). Current treatment modalities include surgical excision, curettage, sclerotherapy, chemical cauterization, electrocautery, and various laser therapies (4).

Surgical excision is traditionally regarded as the standard of care, offering definitive removal of the lesion with a low recurrence rate when complete resection is achieved (5). However, excision may not be feasible for lesions in cosmetically sensitive areas, and it frequently leaves a scar, the size of which depends on the surgeon's precision and lesion location.

Sclerotherapy with polidocanol has emerged as a safe and effective alternative for PG management (6). Given that PG is composed of lobular clusters of capillaries, endothelial damage induced by polidocanol leads to vascular necrosis and subsequent lesion regression. Local intralesional injection of 1% polidocanol has demonstrated cure rates approaching 100%, with good tolerance and a low recurrence rate (7).

Despite the availability of both treatment modalities, direct comparative studies—particularly in pediatric populations—remain scarce. The present retrospective cohort study aimed to compare the efficacy, safety, and patient-reported outcomes of 1% polidocanol sclerotherapy vs. surgical excision in the treatment of pediatric PG. We hypothesized that sclerotherapy would be non-inferior to surgery in terms of cure rates, while offering advantages in procedural simplicity, reduced invasiveness, and superior cosmetic outcomes.

## Methods

This retrospective cohort study was conducted in accordance with the STROBE (Strengthening the Reporting of Observational Studies in Epidemiology) guidelines. Thirty patients diagnosed with pyogenic granuloma (PG) and treated at the Children's Hospital between January 2022 and February 2023 were included. Clinical records were retrospectively reviewed. Ethical approval was obtained from the institutional Research Ethics Committee in January 2024 (approval number: 2023307), and written informed consent was obtained from the parents of all patients.

### Inclusion and exclusion criteria

Eligible patients were (1) aged 0–18 years; (2) clinically diagnosed with PG; and (3) treated with either surgical resection or sclerotherapy at our institution. Patients were excluded if (1) medical records were incomplete; (2) prior treatment for the same lesion had been received elsewhere; or (3) follow-up was less than 12 months.

### Surgical procedure

Surgical excision was performed by one of two attending pediatric surgeons, each with more than 10 years of experience in pediatric surgery, under local or general anesthesia.

### Sclerotherapy procedure

Sclerotherapy was performed by a pediatric surgeons with more than 5 years of experience. Under sterile conditions, 1% polidocanol (Aethoxysklerol®, Chemische Fabrik Kreussler & Co. GmbH, Wiesbaden, Germany) was injected intralesionally using a 30-gauge needle until blanching of the lesion was observed. The maximum injection volume per session was 1–2 mL.

### Outcome assessment

At the end of follow-up, the Patient and Observer Scar Assessment Scale (POSAS) 2.0 was used to evaluate cosmetic outcomes and patient satisfaction. Observer scores were independently assessed by two blinded clinicians. Prior to the study, the observers were calibrated using five external scar images.

### Statistical analysis

Data were analyzed using independent t-tests and *χ*^2^ tests by SPSS software (version 23.0).

## Results

### Clinical characteristics

The cohort consisted of 16 males and 14 females, with a mean age of 7.6 ± 4.1 years (range: 1–13 years). Lesions were most commonly located on the head and neck (*n* = 14, 46.7%) and face (*n* = 12, 40%), followed by the extremities (*n* = 3, 10%) and trunk (*n* = 1, 3.3%). Fifteen patients underwent surgical excision, and 15 received sclerotherapy. Baseline demographic and clinical characteristics were comparable between groups, with no statistically significant differences (Table 1). All patients completed at least 12 months of follow-up, and no cases were lost to follow-up.

**Table 1:** Baseline and procedural-related information in the surgical resection group and the sclerotherapy group.

Characteristics	Surgical resection	Sclerotherapy	Total	*P*-value
Number of cases	15	15	30	
Gender				>0.05
Male	9	7	16	
Female	6	8	14	
Age(years)	9.9 ± 3.3	5.2 ± 3.6		
Anatomical position				>0.05
Face	7	7	14	
Head and neck	6	6	12	
Trunk	1	0	1	
Extremities	1	2	3	
Method of anesthesia				>0.05
General anesthesia	5	7	12	
Local anesthesia	10	8	18	

### Treatment outcomes

All 30 patients achieved complete lesion resolution, yielding an overall cure rate of 100%, with no recurrences observed during follow-up. In the sclerotherapy group, 14 of 15 patients (93.3%) achieved complete resolution after a single session; the remaining patient required a second session for complete clearance. The primary cure rate after one session was slightly lower in the sclerotherapy group than in the surgery group (93.3% vs. 100%), but the difference was not statistically significant. The mean procedure duration was significantly shorter in the sclerotherapy group than in the surgical group (*p* < 0.05; Table 2).

**Table 2:** Comparison of efficacy and cost between surgical resection group and sclerotherapy group.

Characteristics	Surgical resection(n)	Sclerotherapy(n)	*P*-value
Scars	10 (66.6%)	1 (6.66%)	0.02
Depressed	5 (33.3%)	0	
Raised	5 (33.3%)	1 (6.66%)	0.651
Pigmentation	4 (26.6%)	2 (13.3%)	0.693
Postoperative infection	1 (6.66%)	0	
Duration of surgery (min)	18.2 ± 7.1	9.4 ± 3.9	<0.01
Hospitalization expenses (yuan)	5004.83 ± 2424.19	4773.99 ± 1223.05	0.745
Surgery costs (yuan)	1929.45 ± 611.20	1646.60 ± 507.82	0.179
Average times of treatments	1	1.067	
Single session	100%	93.4%	1
Overall cure rate	100%	100%	
Recurrence rate	0%	0%	

**Figure 1:**
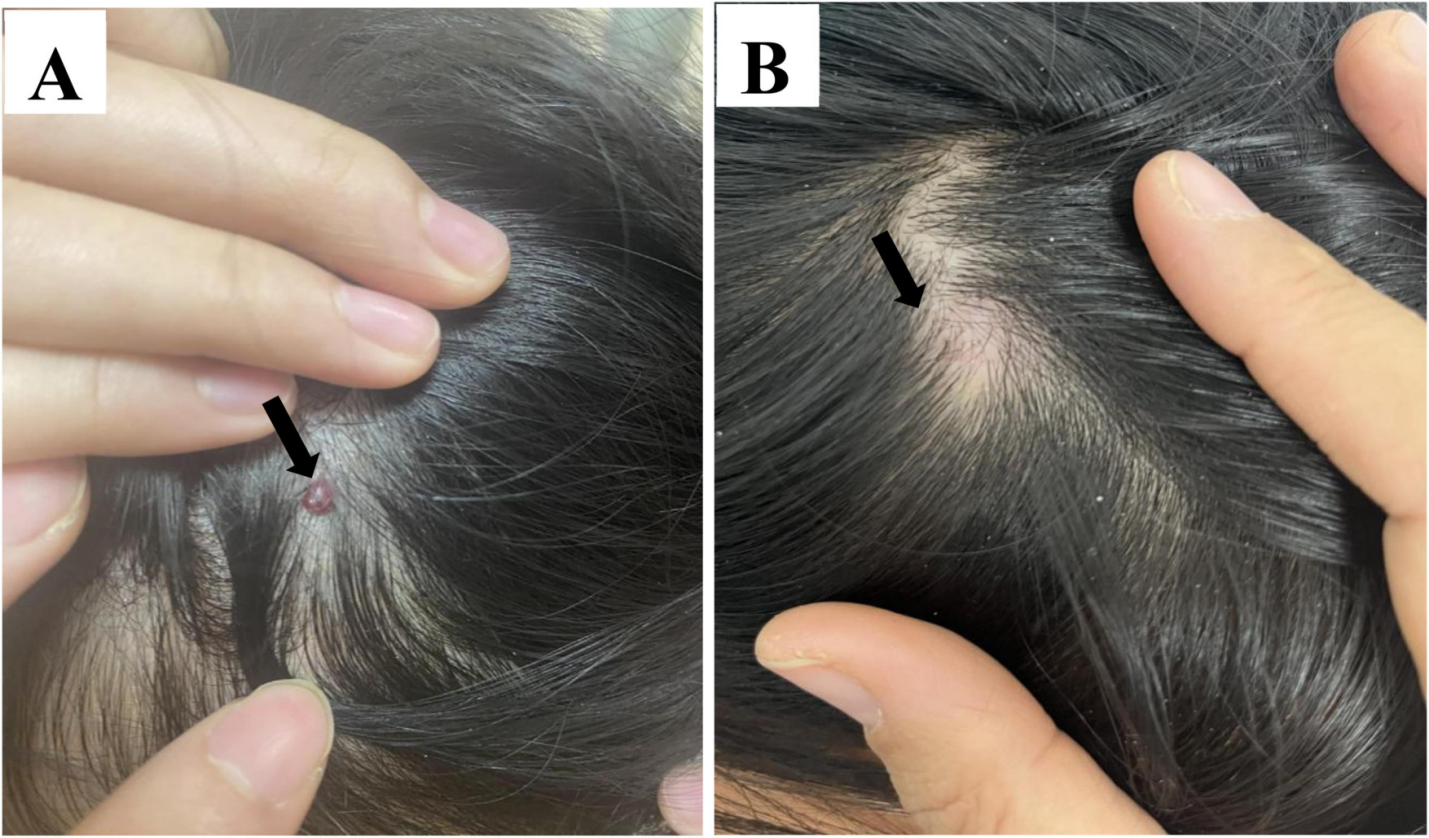
**(A)** pretreatment clinical appearance of the lesion on the scalp, measuring approximately 2 × 3 mm. **(B)** Postoperative view at 6-month follow-up, demonstrating a well-healed linear surgical scar measuring approximately 7 mm in length.

**Figure 2:**
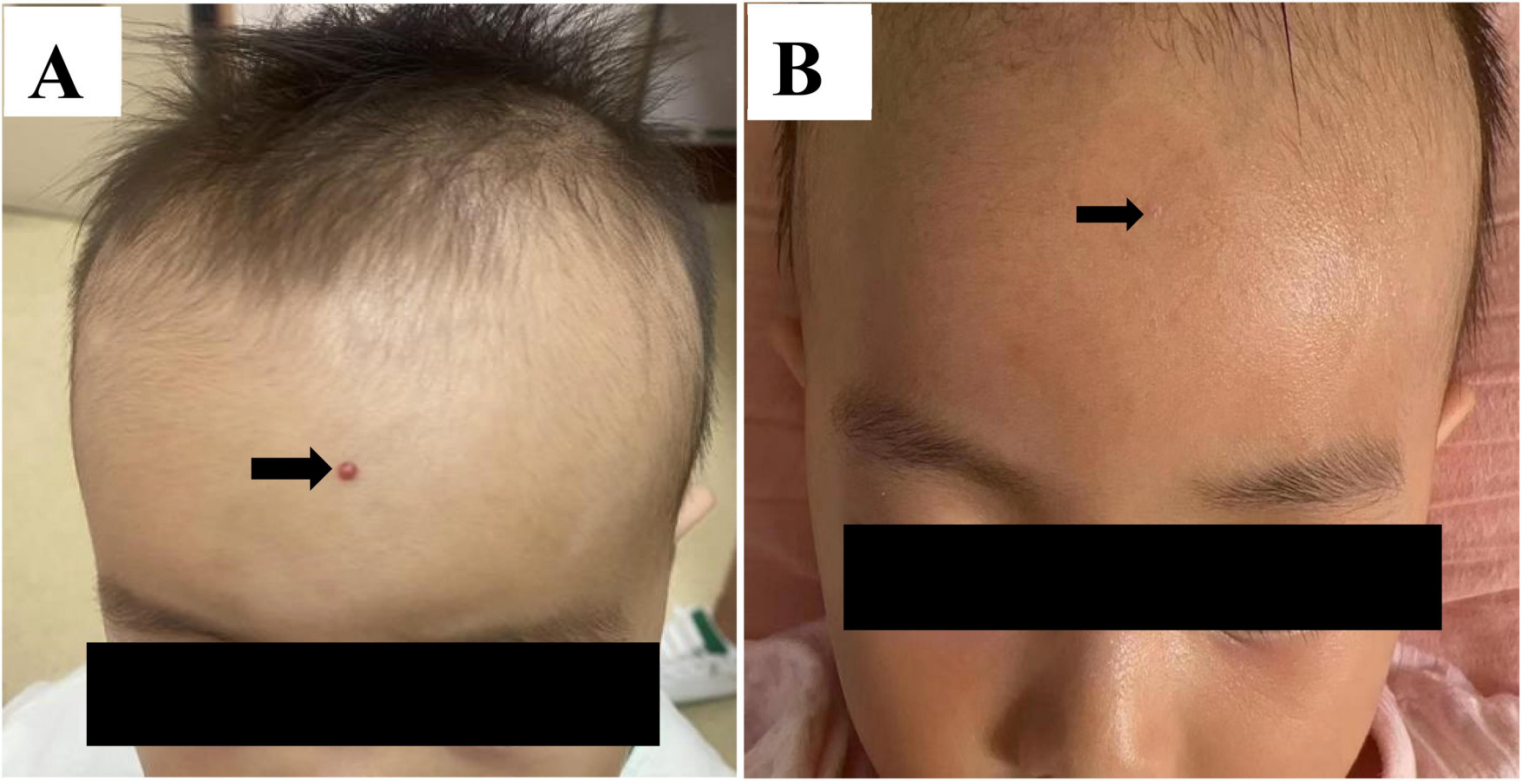
**(A)** Pretreatment clinical appearance of the lesion, measuring approximately 2 × 3 mm. **(B)** Posttreatment view at 6-month follow-up after injection of 0.3 mL of 1% polidocanol, demonstrating complete resolution without significant scarring.

Postoperative scar formation was significantly more frequent in the surgical group (Figure 1) (66.7%, *n* = 10) compared with the sclerotherapy group (6.7%, *n* = 1; *p* < 0.01) (Figure 2). One surgical patient developed a purulent wound infection 3 days after surgery. In the sclerotherapy group, two patients developed transient post-injection erythema; the incidence was not significantly different from the surgical group. No cases of allergic reactions, skin ulceration, or necrosis were recorded. Hospitalization and treatment costs were lower in the sclerotherapy group, although the difference did not reach statistical significance.

### Scar assessment and regression analysis

POSAS 2.0 scores were evaluated by parents and two blinded, calibrated observers. As shown in Table 3, the sclerotherapy group had significantly lower (better) Patient POSAS scores compared with the surgical group (*p* < 0.01). Similarly, Observer POSAS scores were significantly lower in the sclerotherapy group (*p* < 0.01) Table 3.

**Table 3:** Comparison of postoperative satisfaction and POSAS* scores between surgical resection group and sclerotherapy group.

Characteristics	Surgical resection	Sclerotherapy	*P*-value
Postoperative Satisfaction	9.0 ± 1.0	9.4 ± 0.74	0.253
The Patient Scale Of The POSAS	3.7 ± 2.2	1.4 ± 0.50	<0.001
The Observer Scale Of The POSAS	3.5 ± 2.0	1.1 ± 0.35	<0.001
Vascularity	1.13 ± 0.63	0.93 ± 0.25	0.276
Pigmentation	0.26 ± 0.45	0.13 ± 0.35	0.379
Thickness	0.46 ± 0.74	0	0.029
Relief	1.2 ± 1.08	0.66 ± 0.25	0.01
Pliability	0.13 ± 0.35	0	0.164
Surface area	0.33 ± 0.48	0	0.019

* The POSAS can be used in both clinical and research settings in order to measure the effectiveness of scar treatments, monitor scar maturation over time, and identify the need for future treatments. A lower score indicates a less prominent scar. (POSAS 2.0 version scale can be download in http://www.posas.nl).

Despite these differences in POSAS scores, both groups reported high levels of postoperative satisfaction, with no statistically significant difference between treatment groups.

## Discussion

A wide variety of treatment options are available for pyogenic granuloma (PG), including β-adrenergic receptor antagonists, surgical excision, sclerotherapy, laser ablation, electrocautery, curettage, and chemical cauterization (8). Each modality carries unique challenges, including risk of residual vasculature, limited equipment availability, and the need for multiple sessions. Among these, surgical resection is traditionally regarded as the gold standard, offering the lowest recurrence rates (9). However, surgical excision can be technically demanding in anatomically challenging locations such as the oral cavity or distal extremities, and it invariably leaves a scar.

Our results support sclerotherapy with 1% polidocanol as a safe and effective alternative to surgery. We observed a single-session clearance rate of 93.3%, an average of 1.06 treatment sessions per patient, and a 100% overall cure rate, with no recurrences during follow-up. These findings are consistent with previous reports by Carvalho et al. and Li et al., who achieved near-complete resolution using 0.5% and 1% polidocanol, respectively, with minimal recurrence (6). Importantly, we found that sclerotherapy resulted in significantly lower scar incidence and better cosmetic outcomes compared with surgical excision, aligning with the minimally invasive nature of this technique.

Polidocanol induces endothelial injury, thrombosis, and subsequent vessel occlusion, leading to necrosis and detachment of PG lesions. Its anesthetic properties and low incidence of allergic, local, or systemic reactions make it a favorable sclerosing agent compared with other agents (10). In our cohort, no allergic reactions, necrosis, or systemic complications were observed. Nevertheless, the potential for adverse events—including cutaneous ulceration, thrombophlebitis, or nerve injury—should not be overlooked, particularly when excessive volume or concentration is used or when extravascular injection occurs (11). This highlights the importance of proper technique and training to minimize risks.

Despite these promising results, our study has limitations. The relatively small sample size limits the ability to detect rare complications or very low recurrence rates. In addition, the retrospective design may introduce selection bias.

In summary, both surgical excision and sclerotherapy were effective for PG management, but sclerotherapy demonstrated advantages in terms of reduced scarring, shorter procedure duration, and superior cosmetic outcomes. Our findings suggest that 1% polidocanol sclerotherapy may serve as a preferred first-line therapy for pediatric PG. Future large-scale, prospective randomized controlled trials are needed to validate these findings and optimize treatment algorithms.

## Data Availability

The original contributions presented in the study are included in the article/Supplementary Material, further inquiries can be directed to the corresponding authors.
